# The Resistive Switching Characteristics in ZrO_2_ and Its Filamentary Conduction Behavior

**DOI:** 10.3390/ma9070551

**Published:** 2016-07-08

**Authors:** Chun-Hung Lai, Hung-Wei Chen, Chih-Yi Liu

**Affiliations:** 1 Department of Electronic Engineering, National United University, Miaoli 36063, Taiwan; brandon@nuu.edu.tw; 2 Department and Institute of Electronic Engineering, National Kaohsiung University of Applied Sciences, Kaohsiung 80778, Taiwan; cyliu@cc.kuas.edu.tw

**Keywords:** filament, resistance change, top electrode

## Abstract

This study investigated the conduction properties of sputtered ZrO_2_ exhibiting reversible and stable resistance change. Similar current distributions in on/off conduction and set/reset switching were observed in top electrodes with a diameter of 150, 250, and 350 µm. The size independence of current magnitude implied the presence of an uneven filamentary path over the electrode area. Increased current compliance was imposed on the turn-on process, and the observed increase in on-state current and turn-off threshold was attributed to incremental filament diameter. Variations in current conduction and resistance switching were analyzed by monitoring sweeping bias limits in both positive and negative polarities. These experimental observations were interpreted based on the aspect ratio of channels comprising conductive and oxidized filament portions, thereby elucidating the characteristics of filamentary resistive switching.

## 1. Introduction

Resistive random access memory (RRAM), which is a crucial class of nonvolatile memory, is an alternative to commercial floating-gate flash memory [1]. RRAM is advantageous because of its simple device structure, easy operation, and high speed. Bistable low- and high-resistance states (LRS and HRS), can be switched reversibly by applying sweeping or pulse voltages with appropriate magnitude and polarity. The set and reset operations represent transitions to an LRS or HRS, which occur at the switching thresholds of (I_SET_, V_SET_) and (I_RESET_, V_RESET_), respectively. V_SET_ and V_RESET_ are of the same polarity for unipolar resistance switching (URS), whereas opposite polarities are required for bipolar resistive switching (BRS). Previous studies have observed URS and BRS in numerous binary and ternary metal oxides, and the coexistence of two switching modes has been observed in several materials, including TiO_2_ [2], NiO [3], ZnO [4], ZrO_2_ [5], HfO_2_ [6], and Ta_2_O_5_ [7]. ZrO_2_ resistive thin films exhibit long retention and superior endurance in the URS and BRS modes.

Both homogeneous and filamentary types of switching have been proposed in literature. The dependence of current conduction on the electrode area is a key difference between these two switching types. By filament model, the formation of conductive filaments (CFs) in LRS comes from the low-resistance channels between the top and bottom electrodes [8]. In previous studies, spot images have been directly observed by performing probe scanning, which provides strong support for this model [9,10]. An in-between layer was embedded to improve the switching performance by enhancing the confinement of the current path [11,12]. URS and BRS differ in resistance ratio and especially in the abrupt or gradual transition to the HRS [13,14], which implies a specific reset mechanism to be involved in the CF rupture process. The physics and characteristics of CFs provide guidelines for controlling RRAM switching operations and enhancing conduction reliability. In this study, the conduction and switching dispersions in ZrO_2_ devices were examined by applying various current compliances and sweeping voltage ranges. Influence of these measuring conditions was interpreted in perspective of CFs, as compared with those by preparing conditions, e.g., materials, structures, and atmosphere treatment.

## 2. Results and Discussion

**Figure 1:**
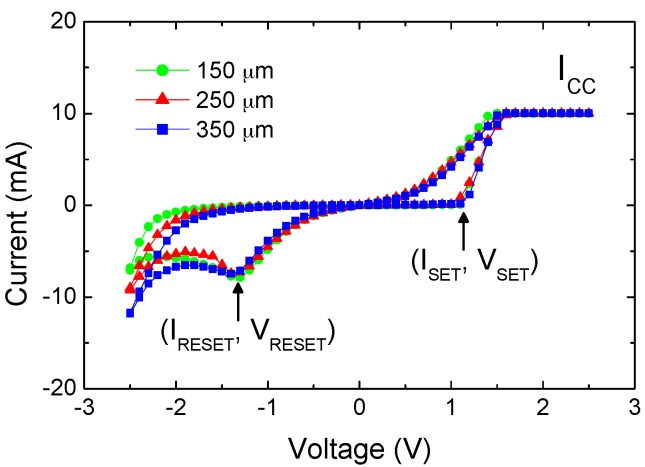
Typical room-temperature I–V curves in linear scale under I_CC_ = 10 mA with top electrode diameter of 150, 250, 350 μm.

The device under test is of the metal-oxide-metal structure, as described in Section 3 by Ti/ZrO_2_/Pt. Figure 1 shows typical I–V sweeps for cells of three top electrode (TE) diameters. All of them exhibited transitions at V_SET_ ≈ +1.1 V and V_RESET_ ≈ −1.2 V after a forming operation at 6 V. Three crucial observations on the BRS polarity, current compliance, and current distribution are detailed as follows. First, a positive V_SET_ polarity indicated that the reduction reaction to the LRS occurred in the ZrO_2_ because of higher potentials at the TE. The Ti electrode functioned as an oxygen ion getter [15], and the positively charged oxygen vacancies remained, thereby forming a low-resistance path. A detailed possible mechanism was suggested by Jeong et al. in terms of electro-chemical reaction involving oxygen ions/vacancies [16]. Once the CF formed at V_SET_, most of the applied current flowed through this path, and that is why I_CC_ was applied to prevent unlimited current rise. Further increases in voltage (i.e., if V_POS_ was higher than V_SET_) and current compliance can create stronger paths or increase the CF diameter. By contrast, V_RESET_ denotes the threshold at which resistance values begin changing. A typical gradual transition to the HRS in the BRS mode manifested as a distinct negative resistance region, which is characteristic of a CF rupture induced by oxidation processes. The extent to which the voltage sweep V_NEG_ surpassed V_RESET_ determined the nature of the turn-off extent. The influence of I_CC_ and V_POS_ on LRS, as well as that of V_NEG_ on HRS, is discussed in subsequent paragraph.

Second, Figure 1 shows the current compliance as a horizontal line in the upper-right corner. This compliance level was applied to prevent current overrise, to control current levels after set-switching, and to monitor the switching mode. Previous studies have adopted current compliance at the sub-milliamp scale for small cell areas (e.g., 1 µm^2^) [13], and even at the nano-amp scale [17]. Proper adoption of down-size cell and compliance level provides a route to the low-power switching operation of ZrO_2_ device. Lee et al. [4] reported a switching mode change from BRS to URS when an I_CC_ of up to 40 mA was applied to a ZnO semiconductor with a TE diameter of 150 µm. The decision to apply I_CC_ values higher than 10 mA in this study was based on the conduction current levels of I_LRS_ and I_HRS_, as well as those obtained at V_SET_ and V_POS_. Under relatively high compliance (e.g., up to 50 mA), the studied ZrO_2_ devices exhibited reproducible and stable BRS properties. The increases in local temperature, known as the Joule heating effect, play a critical role in the electro-thermal process for URS. For the electro-chemical redox in BRS, this thermal impact is not discussed because of the complex nature of material thermal conductivity and filament distribution/density.

**Figure 2:**
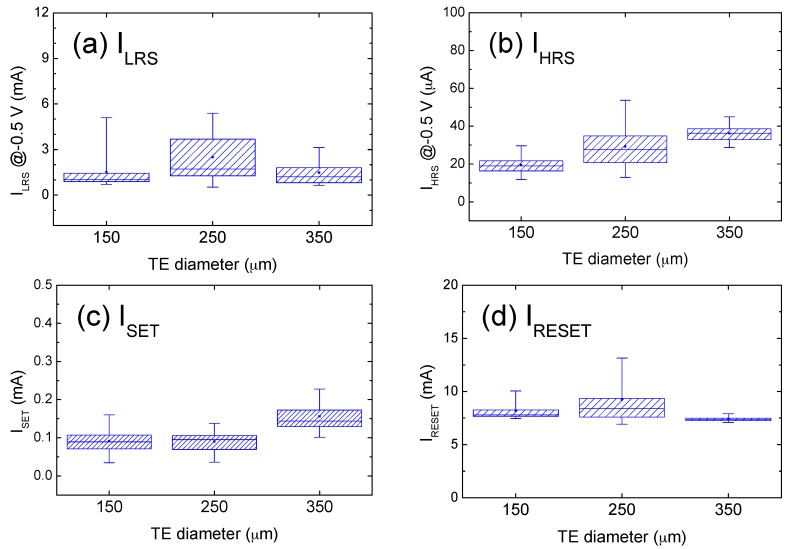
Distribution of critical current parameters extracted from Figure 1 after multiple measurements as a function of electrode areas. (**a**) I_LRS_; (**b**) I_HRS_; (**c**) I_SET_; (**d**) I_RESET_.

As for the third point about the current distribution extracted from Figure 1, Figure 2 shows 100 successive sweeping cycles for conduction currents (I_LRS_, I_HRS_) and switching currents (I_SET_, I_RESET_). LRS and HRS conduction currents (I_LRS_ and I_HRS_) were measured at −0.5 V, at which the I–V curve exhibited a linear relationship. The figures show that I–V curves are independent from TE size by fixing I_CC_ and (V_POS_, V_NEG_). The size-insensitive property of I_LRS_ was attributed to the highly localized filament that formed in the ZrO_2_ matrix [18], which correlated closely with the I_RESET_ threshold for CFs to rupture. Figure 2a,b shows that the current ratio between I_LRS_ and I_HRS_ was approximately two orders of magnitude by 2 mA over 20 µA. Because of the considerably lower I_HRS_ typically observed near zero bias, a larger resistance change ratio exceeding 100 was anticipated for readout voltages less than 0.5 V. Under the condition of V_SET_ ≈ |V_RESET_| and |I_RESET_| > I_SET_, as shown in Figure 2c,d, a larger reset power is required than that at set switching. Rohde [19] observed that the required power was the most dominant parameter for successful switching. The higher power required to initiate a reset transition indicates a more stable conduction in LRS, as compared with that in HRS. Supporting evidence in previous studies includes experimental findings of an improved thermal disturbance immunity facilitated by longer retention in LRS, and the substantially shorter pulse width required to induce a set-switching by an electric pulse [1]. In a compact model for a filament-type RRAM device, the contrasting operational properties were reported, e.g., a gradual resistance change in reset transition while an abrupt change in set switching [20].

**Figure 3:**
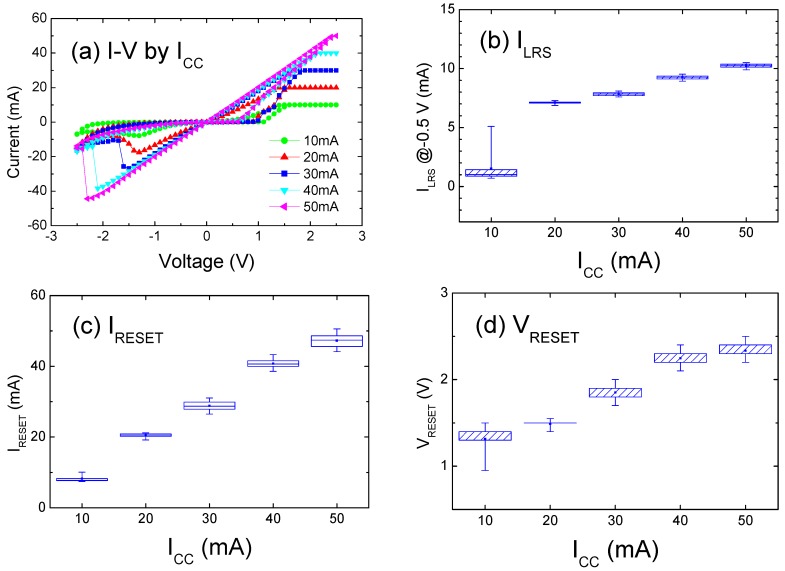
(**a**) Typical room-temperature I–V curves by top electrode (TE) diameter of 150 μm under I_CC_ of 10 to 50 mA; (**b**) Distribution of extracted parameters after multiple measurements for I_LRS_; (**c**) for I_RESET_; and (**d**) for V_RESET_.

Figure 3a shows I–V sweeps as a function of I_CC_, and Figure 3b–d shows the extracted distributions of I_LRS_ and (I_RESET_, V_RESET_). As shown in Figure 3a, higher I_CC_ setting will induce extension on both sides of the I–V curve with clear linearity, indicating the development of an ohmic relationship followed by the CF conduction in LRS. Figure 3b shows the tendency for I_LRS_ to increase in conjunction with I_CC_, which corresponds to the increasing slope of the I–V curve. The corresponding transition is distinctly more abrupt and the switching threshold (I_RESET_, V_RESET_) is higher, as evidenced by the negatively biased I–V curves shown in Figure 3a. Therefore, the distribution tends to increase with I_CC_, as shown in Figure 3c,d. A more detailed explanation based on the filamentary mechanism is given below.

Voltage-induced ion migration and the resulting redox process drive the set transition in the BRS mode [20]. The CF formation is caused by interfacial oxygen ion movement toward the Ti, and also by the resultant local oxygen vacancies accumulated in the ZrO_2_ films [21]. Recent study revealed Ti ion contribution from the TiO_2_ solid electrolyte [22], although Ti electrode migration was rarely reported, as compared with Ag and Cu [23,24]. Yoon el al. [10] declared that the non-uniform and uncontrolled formation of CFs over the cell area is caused by their random growth through the thermally assisted electrochemical reaction. Previous studies have ascribed the correlated increasing I_LRS_ with I_CC_ to the increase in filament diameter or effective area [25,26], although Guan et al. [18] stated that strong filaments with a more favorable percolation of elements are formed under higher I_CC_ values. Noh et al. [20] argued that the filament diameter is controlled by the bias setting of polarity, amplitude, and time. They derived CF resistance as a function of filament diameter based on ohmic conduction. Moreover, McWilliams et al. [27] used equations to demonstrate that I_CC_ correlates explicitly with resistance in the LRS and with I_RESET_. Contrary to the field-induced set process, the reset transition is current driven [5]. Thus, higher I_CC_ leads to rising I_LRS_, and an increasing I_RESET_ typically coincides with an increase in I_LRS_ [27,28]. Rahaman et al. [17] plotted the linear relationship of I_CC_–I_RESET_ and I_CC_–CF diameter. These arguments could be further verified by direct observation of the filaments [29,30].

The increasing I_RESET_ in Figure 3c is approximately equal to I_CC_, and this rise surmounts that of V_RESET_ in Figure 3d. The resultant resistance decline with I_CC_ complies with the resistance decrease in Figure 3b, i.e., the resistance in LRS is related to the resistance at the reset threshold. Furthermore, Wang [31] observed a more abrupt reset process by increasing I_CC_, which similarly occurred at higher V_RESET_. They proposed a scheme of singly connected CFs formed under low I_CC_, whereas net-like CFs generated at high I_CC_. The reset process involves an oxidation reaction caused by oxygen ions to reenter into ZrO_2_; subsequently, a re-oxidized portion forms near the interface. A ruptured filament of shorter length is then connected in series with an HRS region. Park et al. [32] applied a sweeping range to examine the gradual reset transition, and confirmed the existence of incompletely dissolved CFs by performing conductive atomic force microscopy. Long et al. [33] computed reset statistics and confirmed its dependence on I_LRS_ and, therefore, CF size, which was determined by the set or compliance setting in preceding operation. Liu et al. [34] controlled the CF growth process and overcame the intrinsic multistep reset jiggles. The manipulation of set or reset conditions to obtain multiple distinguishable LRSs or HRSs provides a potential RRAM application for multibit storage. Varying the sweeping bias limits is an effective method, as discussed in the next two paragraphs.

**Figure 4:**
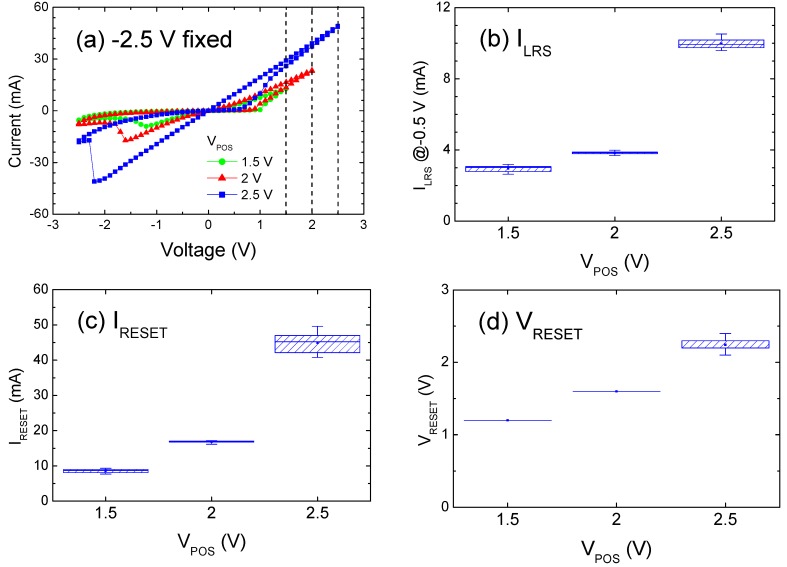

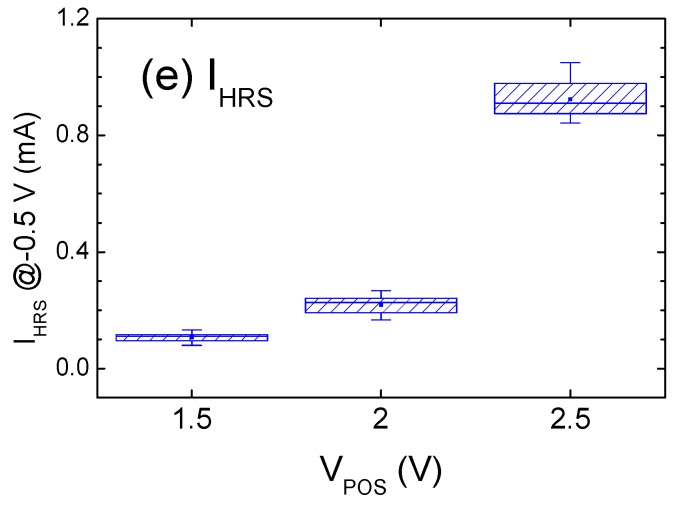
(**a**) Typical room-temperature I–V curves as a function of V_POS_ with no compliance setting; (**b**) Effect of V_POS_ on I_LRS_; (**c**) on I_RESET_; (**d**) on V_RESET_; and (**e**) on I_HRS_.

Because V_SET_ is the threshold at which CFs are formed, it is a reasonable speculation that a sweep value higher than V_SET_ would enhance CF growth. The extent to which V_POS_ is higher than V_SET_ determines the filament diameter or strength. Compliance was removed to examine the V_POS_ effect alone. Figure 4a shows the I–V data where V_POS_ is 1.5, 2, and 2.5 V while −2.5 V is fixed. Similar to Figure 3, Figure 4b–d shows the extracted distributions of I_LRS_ and (I_RESET_, V_RESET_). The impact of V_POS_ on the conduction in LRS is similar to that exerted by I_CC_, including an increase in I_LRS_ and in the steepness of the linear I–V curve, a larger power required at reset, and a more abrupt reset transition. Regarding the relatively small increase in V_RESET_ compared with that of I_RESET_ (see Figure 4c,d, the decrease in resistance at the reset threshold agrees with the decreasing resistance in LRS (see Figure 4b). The similarity between the effects of I_CC_ and V_POS_ was ascribed to the increase in CF diameter. Excess bias expels additional oxygen ions to the Ti reservoir and leaves an oxygen-deficient region with an enhanced cross-section. When the CFs ruptured under the same magnitude (i.e., −2.5 V) to have an identical re-oxidized length, the remaining CFs still exhibited lower resistance under the preceding V_POS_ values, which explains why the distribution in Figure 4e shows an increase in I_HRS_ as V_POS_ increases.

**Figure 5:**
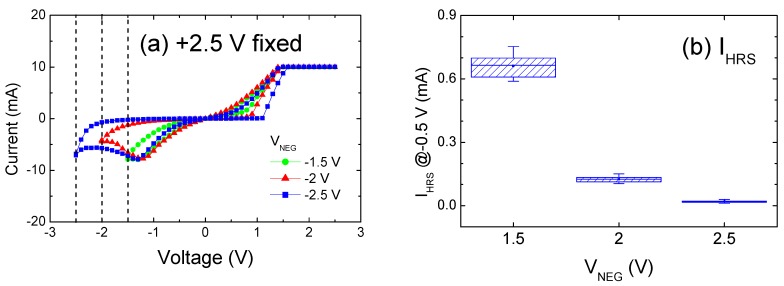
(**a**) Typical room-temperature I–V curves as a function of V_NEG_ under I_CC_ of 10 mA; (**b**) Effect of V_NEG_ on I_HRS_.

Figure 5a shows the I–V sweeps under various V_NEG_ settings. The dotted lines denote −1.5, −2, and −2.5 V, with I_CC_ maintained at +2.5 V. Contrary to the CFs growth as a result of the V_POS_ extent, V_NEG_ influenced the extension of the re-oxidized segment after reset operation. The decreasing I_HRS_ shown in Figure 5b is primarily attributed to the highly resistive re-oxidized portion, or could be ascribed to the shortened length of partially disconnected or narrower CFs. Numerous previous studies have reported that controlling the sweeping range in the reset polarity reduces the value of I_HRS_ [21,26,35,36,37]. Park et al. [35] successfully modulated I_HRS_ and therefore demonstrated the feasibility of multibit applications by adjusting the value of V_NEG_. An engineered Schottky barrier height between Ir and TiO_X_ explains this effect. Yu et al. [36] ascribed the decrease in I_HRS_ to a large tunneling gap between the electrode and residual CFs, and proposed that the I_HRS_–V_NEG_ mechanism is associated with the switching mode and electrode contact. In summary, the variation in the aspect ratio of the filament model presented in this study intuitively explains the difference between the values shown in Figure 4e and Figure 5b; i.e., I_HRS_ increases in conjunction with the CF diameter, whereas I_HRS_ declines in conjunction with CF length.

## 3. Materials and Methods

Sputtered ZrO_2_ (approximately 60-nm thick) was deposited on a Pt/Ti/SiO_2_/Si substrate at 250 °C and 10 mTorr under an O_2_: Ar gas mixture with a ratio of 6:12 and a flow rate of 18 cm^3^ per minute. Because of its oxygen getter properties, Ti was sputter-deposited as the top electrode (TE), and then patterned using a shadow mask with various diameters (150, 250, and 350 µm) to analyze the size effect. The devices were tested in tri-layer structures comprising Ti (110 nm)/ZrO_2_ (60 nm)/Pt. Current-voltage (I–V) curves were obtained using an HP4155A semiconductor parameter analyzer. Device switching between the LRS and HRS was achieved by applying a direct voltage sweep mode while the bottom electrode was grounded. Bias was applied to the TE during each sweep cycle in the range of V_POS_ to −V_NEG_, which denote the positive and negative bias limits, respectively. The current compliance I_CC_ was set to 10–50 mA at the V_SET_ side to control the current level in the LRS. To differentiate between the dependence of specific parameters clearly, this study refers only to their magnitudes and ignores the corresponding signs. Setting V_POS_ (V_NEG_) higher than V_SET_ (V_RESET_) is necessary for successful switching. Ambient water vapor pressure is kept constant to exclude the effect of moisture on the switching characteristics [38,39]. Unless stated otherwise, the data for the I–V curves were measured at room temperature, with an I_CC_ of 10 mA, TE diameter of 150 µm, and a sweeping range of ±2.5 V.

## 4. Conclusions

The effect of current compliance and sweeping range on sputtered Ti/ZrO_2_/Pt was investigated by examining the distributions of the corresponding I–V curves. The area independence of conduction and switching currents indicates that the formation of CFs was localized over the electrode. The on-state current and corresponding turn-off threshold increased in conjunction with the current compliance. Similar results were observed when higher V_POS_ values were applied. The formation of CFs with increased diameters explains these experimental observations. By contrast, extending V_NEG_ resulted in shorter CFs and a re-oxidized portion, thereby decreasing the I_HRS_.
